# Atypical Presentations of Myasthenia Gravis as Acute Respiratory Failure: A Rare Case

**DOI:** 10.7759/cureus.67635

**Published:** 2024-08-23

**Authors:** Bingu Shiv Kiran Reddy, Ulhas Jadhav, Pankaj Wagh, Jay Bhanushali, Souvik Sarkar

**Affiliations:** 1 Respiratory Medicine, Jawaharlal Nehru Medical College, Datta Meghe Institute of Higher Education and Research, Wardha, IND

**Keywords:** achr antibody, neuromuscular junction disorders, dyspnea, autoimmune neuromuscular disease, muscle weakness

## Abstract

Myasthenia gravis (MG) is a chronic neuromuscular disease characterized by the progressive weakness of voluntary muscles, which encompass those in the face, throat, diaphragm, and those attached to bones. Patients commonly present with specific muscle weakness, such as difficulty in eye movement, facial expression, or swallowing, rather than generalized fatigue. However, as the disease advances, the majority of patients develop respiratory symptoms, which can significantly impact their quality of life. This makes the management of respiratory comorbidities essential, as respiratory tract infections can lead to exacerbations of MG and trigger a myasthenic crisis, necessitating immediate medical intervention. This report highlights a patient who initially presented with acute respiratory distress and was subsequently diagnosed with MG, underscoring the importance of recognizing respiratory symptoms in the context of this condition.

## Introduction

Myasthenia gravis (MG) is an autoimmune illness, postsynaptic neuromuscular junction disease caused by the production of antibodies directed against the postsynaptic membrane. Most nations have a prevalence of 250 per million for MG, with an annual incidence of 20 per million. Antibodies against postsynaptic extracellular domains of the nicotinic acetylcholine receptor (AChR) are present in the most prevalent kind of MG. A total of 10%-15% of MG patients do not have an AChR antibody [1].

Out of all the MG patients, 80% experience broad muscle weakness, whereas only 20% have weakness restricted to their ocular muscles. Respiratory muscle involvement is possible in such cases since laryngeal and pharyngeal functions depend on these muscles. Variations in muscle weakness throughout the day and with repeated use are typical for MG, with symptoms worsening with continued muscle use [2]. The specificity of autoantibodies and the differentiation between localized and widespread symptoms are used to identify MG subtypes. Additional requirements for the MG subgroups include age of onset and thymus pathology. A total of 10% of MG patients have muscle weakness and AChR antibodies due to a thymoma [3].

## Case presentation

A 41-year-old female presented to the emergency room with complaints of cough with expectoration for seven days and shortness of breath which was insidious in onset and gradually progressive Modified Medical Research Council (MMRC) grade IV over four days and fever of 38.6°C from the last few days. The patient also complained of dysphagia to solids and liquids for two days. The patient was diagnosed with hyperthyroidism three months back.

**Figure 1:**
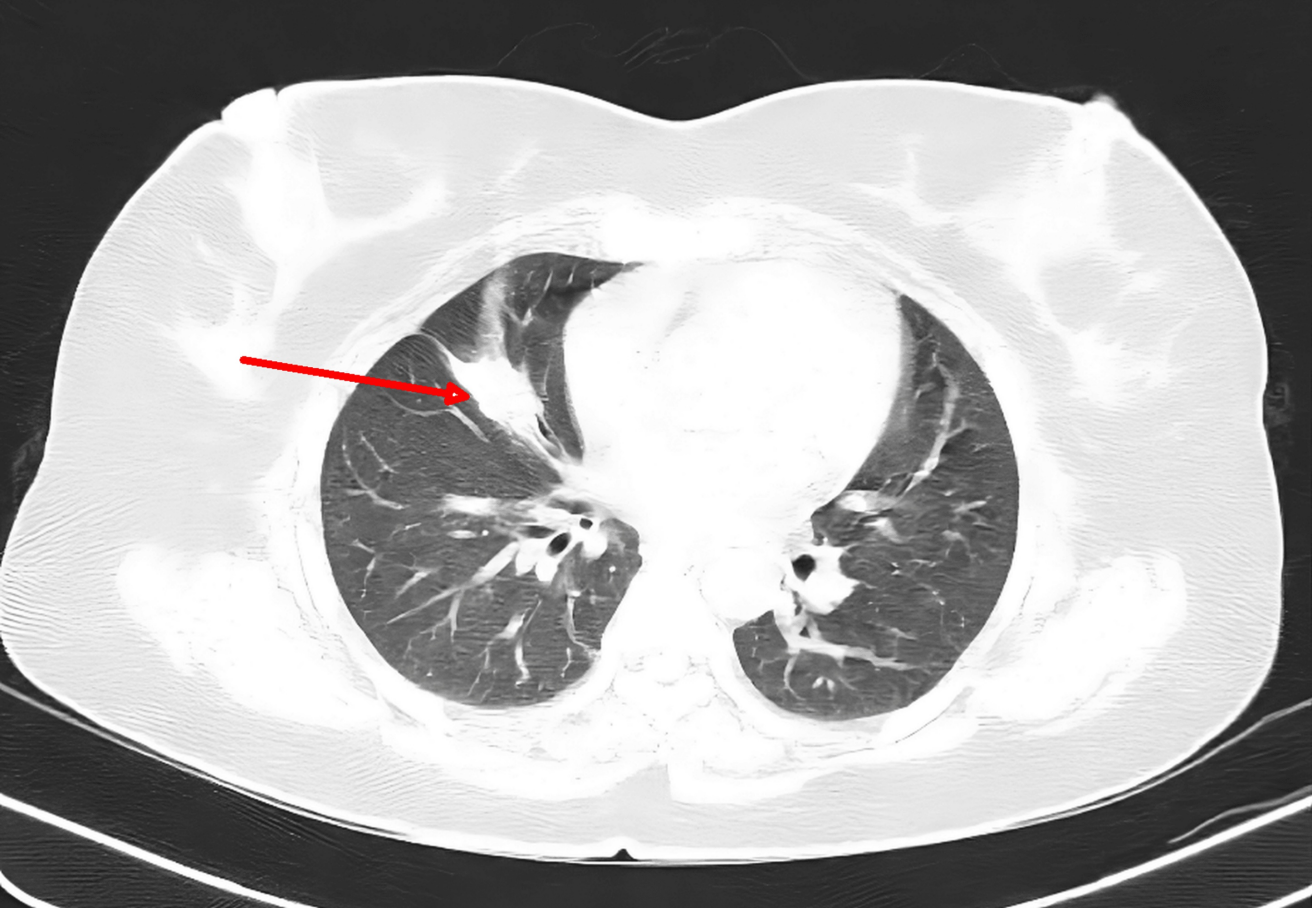
HRCT thorax showing segmental atelectasis HRCT: High-resolution computed tomography

**Figure 2:**
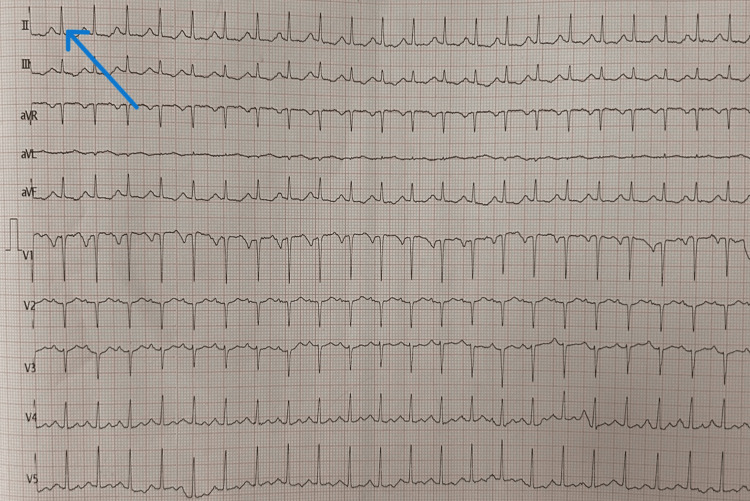
ECG suggestive of sinus tachycardia (arrow) ECG: Electrocardiogram

The patient was conscious, oriented, obeying commands, and well-built. She was breathless and unable to complete sentences in one breath. Pulse was 124/min and regular; blood pressure was 140/100 mmHg; respiratory rate was 32/min with the use of accessory muscles; oxygen saturation (SpO_2)_ was 73% on room air; white blood cell count (WBC) was 9000/cumm; and fever, pallor, icterus, clubbing, cyanosis, lymphadenopathy, and edema were absent on general examination. Respiratory system examination revealed bilateral breath sounds with no adventitious sounds. Central nervous system examination showed a Glasgow Coma Scale of E4M6V5, moving all four limbs. Ultrasonography (USG) of the neck was done which revealed a well-defined hyperechoic nodule in the isthmus measuring 0.3 x 0.4 cm and in the left lobe of the thyroid measuring 0.3 x 0.6 cm. Chest X-ray was done which was suggestive of no obvious pleuroparenchymal disease. High-resolution computed tomography (HRCT) (Figure 1) revealed a segmental atelectasis (major fissure on the right side). Videolaryngoscopy(VDL) was performed to rule out upper airway obstruction, and it was suggestive of mildly inflamed glottis but otherwise normal with no obstruction or foreign body. The patient was started on appropriate medication for the same. Bronchoscopy was done after which a bronchoalveolar lavage sample was sent for cytopathology which revealed a blood-mixed inflammatory bronchial smear. Electrocardiogram (ECG) revealed sinus tachycardia (Figure 2). 

Neurological opinion was taken in view of restrictive respiratory movement to rule out the neuromuscular disorder, after which a nerve conduction study was done on which compound muscle action potential (CMAP) amplitude could not be elicited in the left median, ulnar bilateral tibial, and peroneal nerves. Normal sensory nerve action potential (SNAP) amplitude could not be elicited in the left median and ulnar nerves which finally revealed sensory-motor polyneuropathy. The anti-AChR receptor antibody was found to be 35.4 nmol/L and the anti-musk antibody 0.11 post to which the patient was started on pyridostigmine tablets. The patient was further admitted to the intensive care unit and was immediately given a trial of noninvasive ventilation with oxygen of 100%, inspiratory pressure of 20 cm of H_2_O and positive end expiratory pressure of 5 mmHg given respiratory distress.

The study of arterial blood gas revealed a partial pressure of 0_2 _of 45 mmHg hypoxia, which was suggestive of type 1 respiratory failure (PC0_2_ 39 mmHg, pH 7.38). The patient was started on appropriate treatment for the same. The patient responded well to the treatment, and bilevel positive airway pressure was gradually weaned off. A pulmonary function test was carried out, and it was suggestive of restrictive lung disease. 

## Discussion

MG exhibits a bimodal age distribution, with an early peak in the second or third decade and a late peak in the sixth or eighth decade. This autoimmune disorder is characterized by varying degrees of muscle weakness, which can affect different muscle groups. Approximately 15% of cases involve the ocular muscles, leading to symptoms such as ptosis and diplopia, while 50% of cases involve the bulbar muscles, impacting speech, chewing, and swallowing. Additionally, the condition can affect the respiratory and limb muscles [4].

Weakness gets worse at night or after working out. Approximately 15% of patients have bulbar symptoms, such as fatigue, chewing, dysphagia, and dysarthria. Respiratory insufficiency can result from the involvement of the muscles of breathing, even if separate respiratory muscular weakness is a less common occurrence. Although it has not been frequently reported, respiratory dysfunction as the initial clinical manifestation of MG has not been precisely quantified by an ad hoc investigation. Despite the fact that respiratory failure in the setting of MG can be quite serious, in our situation, dyspnea predominated during exertion, and hypoxemia was never dangerous. It is likely that MG in our case was latent or pauci-symptomatic. The patient may have had an upper respiratory tract infection and then had respiratory muscle weakness from MG, based on symptoms including fever at the outset of the clinical presentation [5].

The main therapies for severe MG exacerbations are plasma exchange and intravenous immunoglobulin. For the majority of MG patients, high-dose corticosteroids, complement inhibitors, and neonatal fragment crystallizable receptor (FcRn) blockers are examples of fast-acting therapies that work.

Our case highlights the significance of taking neurological factors into account in the acute context when dyspnea cannot be attributed to cardiac or respiratory causes. When acute respiratory failure occurs, emergency physicians must promptly recognize MG and include it in the differential diagnosis.

## Conclusions

This case of a 41-year-old female presented with severe respiratory distress, dysphagia, and progressive shortness of breath which was later identified as MG and restrictive lung disease. This case highlights how important it is to consider neurological variables when dyspnea cannot be explained by cardiac or respiratory problems in acute situations. Emergency clinicians need to quickly identify MG and include it in the differential diagnosis when acute respiratory failure occurs, as prompt recognition and treatment are crucial for improving patient outcomes.

## References

[1] Beloor Suresh A, Asuncion RM (2024). Myasthenia Gravis. https://www.ncbi.nlm.nih.gov/books/NBK559331/.

[2] Gilhus NE (2023). Myasthenia gravis, respiratory function, and respiratory tract disease. J Neurol.

[3] Hansen JS, Danielsen DH, Somnier FE, Frøslev T, Jakobsen J, Johnsen SP, Andersen H (2016). Mortality in myasthenia gravis: a nationwide population-based follow-up study in Denmark. Muscle Nerve.

[4] Oosterhuis HJ (1989). The natural course of myasthenia gravis: a long term follow up study. J Neurol Neurosurg Psychiatry.

[5] Vaidya H (2006). Case of the month: unusual presentation of myasthenia gravis with acute respiratory failure in the emergency room. Emerg Med J.

